# Frontier and hot topics in home enteral nutrition based on CiteSpace bibliometric analysis

**DOI:** 10.3389/fnut.2024.1386777

**Published:** 2024-06-20

**Authors:** Shuwen Qin, Qin Chen, Jingjing Huang, Dandan Xu, Kangkang Hu, Yaxi Suo, Qin Hu, Yangyao Peng

**Affiliations:** ^1^Department of Neurosurgery, Zhongnan Hospital of Wuhan University, Wuhan, China; ^2^Department of Hematology, Zhongnan Hospital of Wuhan University, Wuhan, China; ^3^Department of Plastic Surgery, Zhongnan Hospital of Wuhan University, Wuhan, China; ^4^Department of Critical Care Medicine, Zhongnan Hospital of Wuhan University, Wuhan, China

**Keywords:** CiteSpace, bibliometric analysis, home enteral nutrition, management, HEN

## Abstract

**Background:**

The benefits of home enteral nutrition (HEN) are increasingly recognized, with more scholars focusing on this field. This study aimed to comprehensively identify collaborative networks, analyze, and track research trends, focus on current hotspots, and accurately predict the forefront and focus of home enteral nutrition.

**Methods:**

A computer search of the Web of Science Core Collection (WoSCC) was conducted for studies related to home enteral nutrition published from January 1, 2004, to December 31, 2023, and select them in compliance with the PRISMA guidelines. The CiteSpace software was used for bibliometric visualization and comparative analysis of countries, institutions, journals, references, and keywords.

**Results:**

A total of 1,113 documents were included, showing a steady annual increase in publication volume. The United States and the Mayo Clinic were the top publishing country and institution, with 302 and 41 papers, respectively. “CLIN NUTR” had the highest number of publications, totaling 221, while “ESPEN guideline on home enteral nutrition” was the most cited reference, with 43 citations. The most prolific author was Manpreet S with 29 papers.

**Conclusion:**

The management of HEN is a current research hotspot. The safety of HEN and how to improve patient compliance are critical areas for researchers to consider. Future research could focus on these aspects. The blurring of boundaries between hospital and home care and how to utilize telemedicine technologies to serve more patients deserve in-depth exploration. Researchers worldwide should combine their unique characteristics and advantages to strengthen international cooperation.

## 1 Introduction

Home Enteral Nutrition (HEN) is a form of enteral nutrition support conducted in non-hospital settings such as home and community care facilities under the guidance of a specialized nutrition support team (1). HEN is primarily suitable for patients with essentially normal gastrointestinal function but are unable to achieve sufficient nutritional intake through oral routes due to conditions such as swallowing disorders caused by neuro-muscular dysfunction or obstructions resulting from head and neck or upper gastrointestinal tumors (2, 3). The implementation of HEN not only improves the nutritional status of patients, reduces hospitalization time and medical costs, increases bed turnover rates, but also enhances the quality of life for patients while reducing psychological stress and caregiving burden for caregivers (3, 4).

The 2020 guidelines on HEN released by the European Society for Parenteral and Enteral Nutrition (ESPEN) emphasized the need for ongoing dynamic monitoring by a specialized nutrition support team during the process of implementing home nutrition support therapy (2). The guidelines stressed the importance of adjusting nutritional plans based on the specific conditions of patients and promptly identifying and addressing complications associated with HEN. With the continuous maturation of HEN technology and its increasingly apparent advantages, the global population relying on long-term HEN has rapidly expanded.

Despite the effectiveness of HEN in promoting the recovery of gastrointestinal function and enhancing the immune system, practical challenges persist. Variability in self-management skills among patients and caregivers, coupled with the lack of a comprehensive community healthcare service system, often leads to a range of complications in patients receiving HEN, such as diarrhea, gastroesophageal reflux, aspiration, tube occlusion, rupture, displacement, and nutritional intolerance (5–7). To address these issues, the academic community has conducted numerous studies related to HEN in recent years. Despite its importance, navigating the vast literature and data on HEN practices and outcomes can be challenging due to the complexity and volume of available information. Historically, this has led to gaps in the systematic understanding and optimization of HEN practices.

As research on HEN rapidly evolves, there is a need for a deeper understanding of the development process and research trends in this field. Bibliometrics, a mathematical and statistical tool for quantitatively analyzing all knowledge, has been employed to assess distribution, collaboration, citations, keywords, hotspots, and frontiers (8, 9). CiteSpace is visualization software tools used for bibliometric analysis (10, 11). Network maps generated by this software tool allows researchers to intuitively analyze the status of the field, identify research hotspots and emerging trends. Visual analytics in healthcare allow for the effective synthesis of large datasets, providing insights not easily discerned through traditional data analysis methods. By applying these techniques to HEN, our research identified trends, patterns, and anomalies in the data, making significant contributions to academic research and clinical practice. For instance, visualization tools can help clarify the relationship between patient outcomes and specific HEN protocols, highlight geographic and demographic differences in HEN usage, and provide a clearer understanding of the progress of HEN techniques and methodologies over time. Furthermore, the use of visual analysis meets the critical need for accessible, understandable, and actionable information that can support clinicians, researchers, and policymakers in making evidence-based decisions. It also promotes better patient education and engagement by providing a clear visualization of how different HEN strategies lead to different health outcomes. In summary, our work not only fills existing gaps by enhancing the granularity and clarity of data interpretation in the HEN field but also lays the foundation for future research that can further utilize visual analysis to foster understanding and improve practices in the home care setting.

Therefore, this study utilizes CiteSpace software for analysis. Literature related to HEN from 2004 to 2023 is retrieved, and based on knowledge mapping, the study aims to analyze research hotspots and emerging trends, providing specific directions for future research in this field.

## 2 Materials and methods

### 2.1 Data acquisition and search strategy

This study chose to utilize the Web of Science Core Collection (WoSCC) as the data source. This database encompasses a vast array of interdisciplinary, high-impact, international, and comprehensive academic journals. This selection has garnered widespread recognition among numerous researchers. Moreover, there is evidence suggesting that, when conducting visual analysis using CiteSpace, the WoSCC database can yield superior knowledge map effects (12, 13). This database is widely regarded as the optimal choice for conducting literature analysis. Therefore, we reasonably and effectively selected the Web of Science database as the source of data for our study.

The literature search in WoSCC was conducted within a one-day time frame, specifically up to January 1, 2024. To mitigate any biases arising from database updates, the search was completed within the same day. We employed the following search terms: the subject terms were “home enteral nutrition,” “home enteral “feeding,” or “home EN.” To analyze the status, hot topics, and frontier trends more accurately in HEN research, the search time span was set from January 1, 2004, to December 31, 2023. The literature types were restricted to “ARTICLE” or “REVIEW” the language was limited to English to avoid geographical distribution effects on the results.

The content of literature records, including “full record and cited references,” was downloaded and saved in plaintext file formats to ensure the integrity and availability of the data.

### 2.2 Statistical analysis

A bibliometric analysis was conducted on 1,186 documents related to HEN using CiteSpace (version 6.2.R4), a tool renowned for its precise literature review capabilities. CiteSpace leverages multivariate, time-dependent, and dynamic visualization techniques to automatically generate visual maps. These maps are instrumental in identifying research hotspots and trends within specific time frames and scientific domains (14). The visual network diagrams produced by CiteSpace consist of nodes and links, where each node represents an entity, such as an author, an institution, or a country. The connections between different nodes illustrate a network of relationships involving collaboration, co-citation, or co-occurrence, with thicker lines indicating more significant collaboration (15). A node’s centrality, as denoted by a larger circle and a higher centrality score, signifies its importance within the domain (16).

The analysis incorporated literature data spanning from January 2004 to December 2023, with a temporal resolution of 1 year and a threshold set to the top 50 entities for selection. The “pathfinder network” and “crop network for each slice” functions were utilized to refine the output diagrams. Nodes were designated based on countries, institutions, authors, keywords, co-citations of journals, and co-citations of references, facilitating the construction and analysis of a knowledge map for HEN research. Data management, chart creation, and table formulation were performed using Microsoft Excel 2021 software.

## 3 Results

### 3.1 Annual output and categories

**Figure 1:**
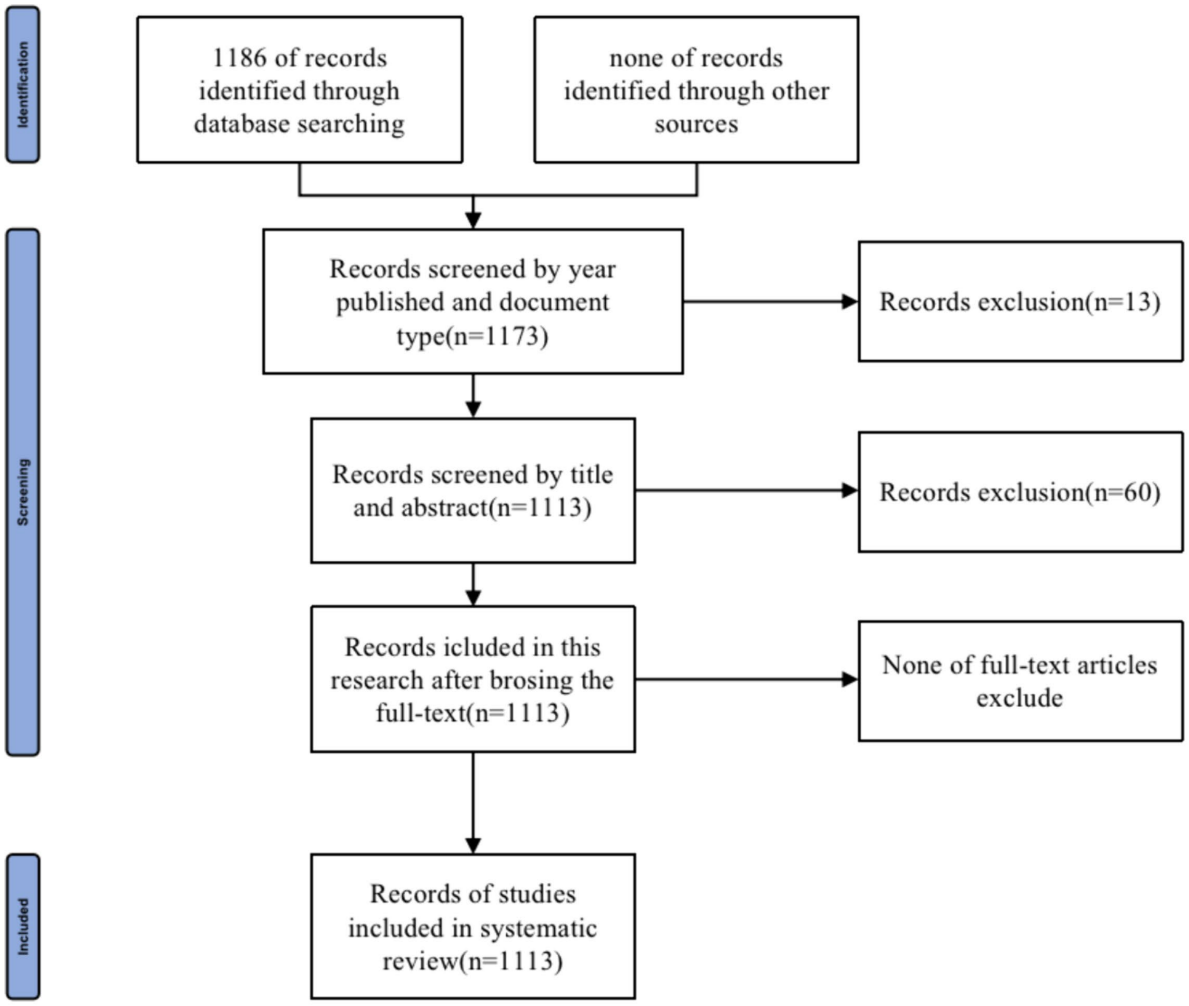
The flowchart searching papers in databases.

**Figure 2:**
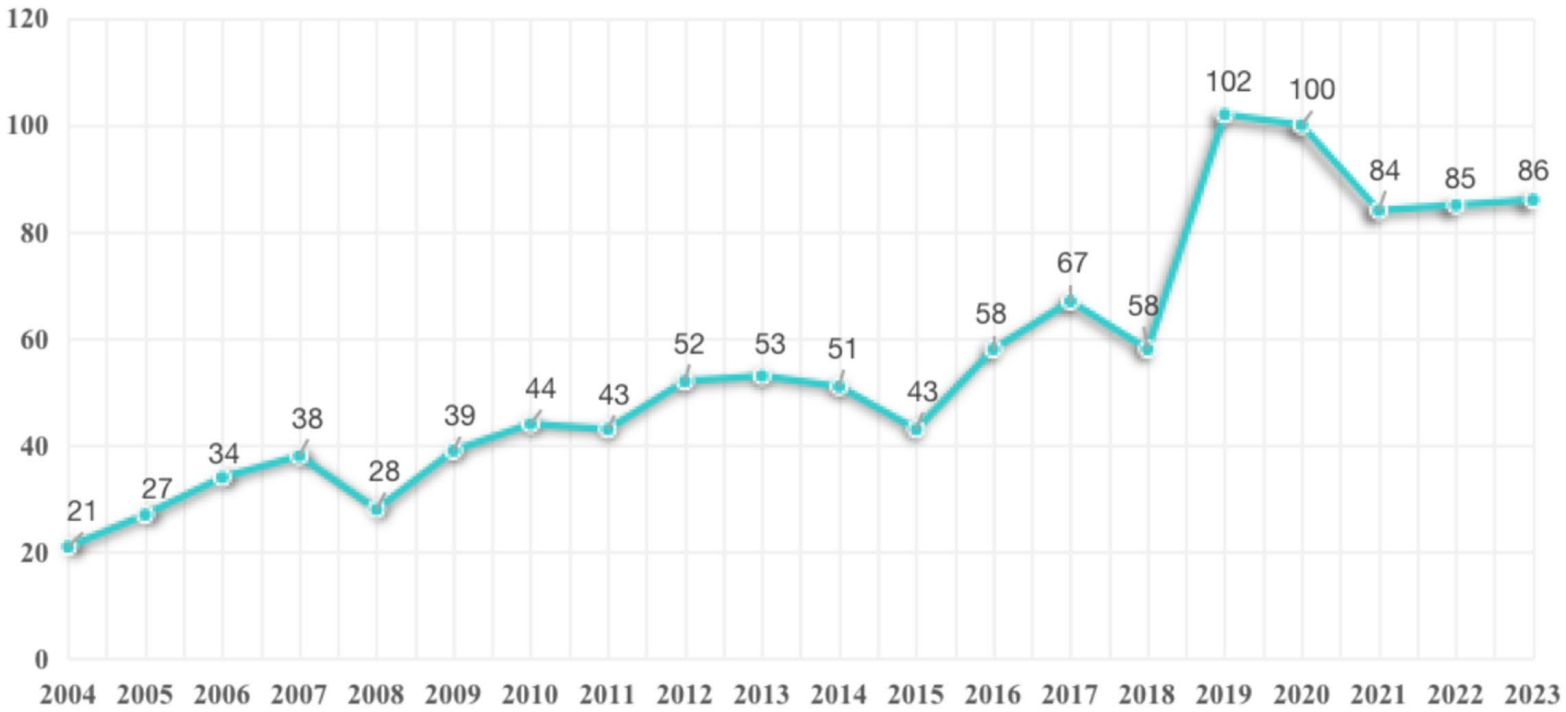
The annual number of publications on HEN from 2004 to 2023.

By searching the WoSCC database, a preliminary review identified a total of 1,186 documents related to HEN from 2004 to 2023. Based on the year of publication and type of document, 13 studies were excluded. After initially screening titles and abstracts, 60 documents were removed. The remaining 1,113 publications were not excluded after full-text analysis. Ultimately, 1,113 studies met the inclusion criteria. The literature screening process was illustrated in Figure 1. The changes in annual publication volume not only reflect the activity level in this field but also highlight the degree of attention to specific research topics. As revealed in Figure 2, although the number of HEN-related publications has fluctuated annually over the past 20 years, there has been an overall upward trend. This indicates that the HEN field has gradually attracted more scholars’ attention in recent years and has become a research hotspot.

Over the course of these 20 years, the development of the HEN literature can be divided into three distinct phases: from 2004 to 2018 was the initial steady growth period, followed by a rapid development period from 2018 to 2020, and then a relatively stable growth phase from 2018 to 2023. During this time, the average annual publication volume was 59 papers, with the lowest volume occurring in 2004 at just 24 papers, and the highest volume in 2019, reaching 105 papers. This trend and data highlight the development and changes in the HEN research field over time, as well as the growing interest in this field within the academic community.

### 3.2 Analysis of journals

From 2004 to 2023, the top five journals in terms of the number of publications are as follows: “NUTRITION IN CLINICAL PRACTICE” with 105 articles, accounting for 8.85%; “JOURNAL OF PARENTERAL AND ENTERALNUTRITION” with 101 articles, accounting for 8.52%; “NUTRICION HOSPITALARIA” with 85 articles, accounting for 7.17%; “CLINICAL NUTRITION” with 49 articles, representing 4.13% of the total number of papers; and “NUTRIENTS” with 46 articles, making up 3.88%. Table 1 provides detailed information about other major publishing journals.

**Table 1:** The top 10 journals on HEN from 2004 to 2023.

Ranking	Journal	Count	Centrality	IF
1	NUTRITION IN CLINICAL PRACTICE	105	0.01	3.1
2	JOURNAL OF PARENTERAL AND ENTERALNUTRITION	101	0.01	3.4
3	NUTRICION HOSPITALARIA	85	0.02	1.2
4	CLINICAL NUTRITION	47	0.01	6.3
5	NUTRIENTS	46	0.03	5.9
6	JOURNAL OF HUMAN NUTRITION ANDDIETETICS	24	0.02	1.4
7	NUTRITION CLINIQUE ET METABOLISME	24	0.05	0.8
8	CLINICAL NUTRITION ESPEN	17	0.07	3.0
9	NUTRITION	17	0.02	4.4
10	JOURNAL OF PEDIATRIC GASTROENTEROLOGY AND NUTRITION	15	0.05	2.9

### 3.3 Analysis of countries

**Figure 3:**
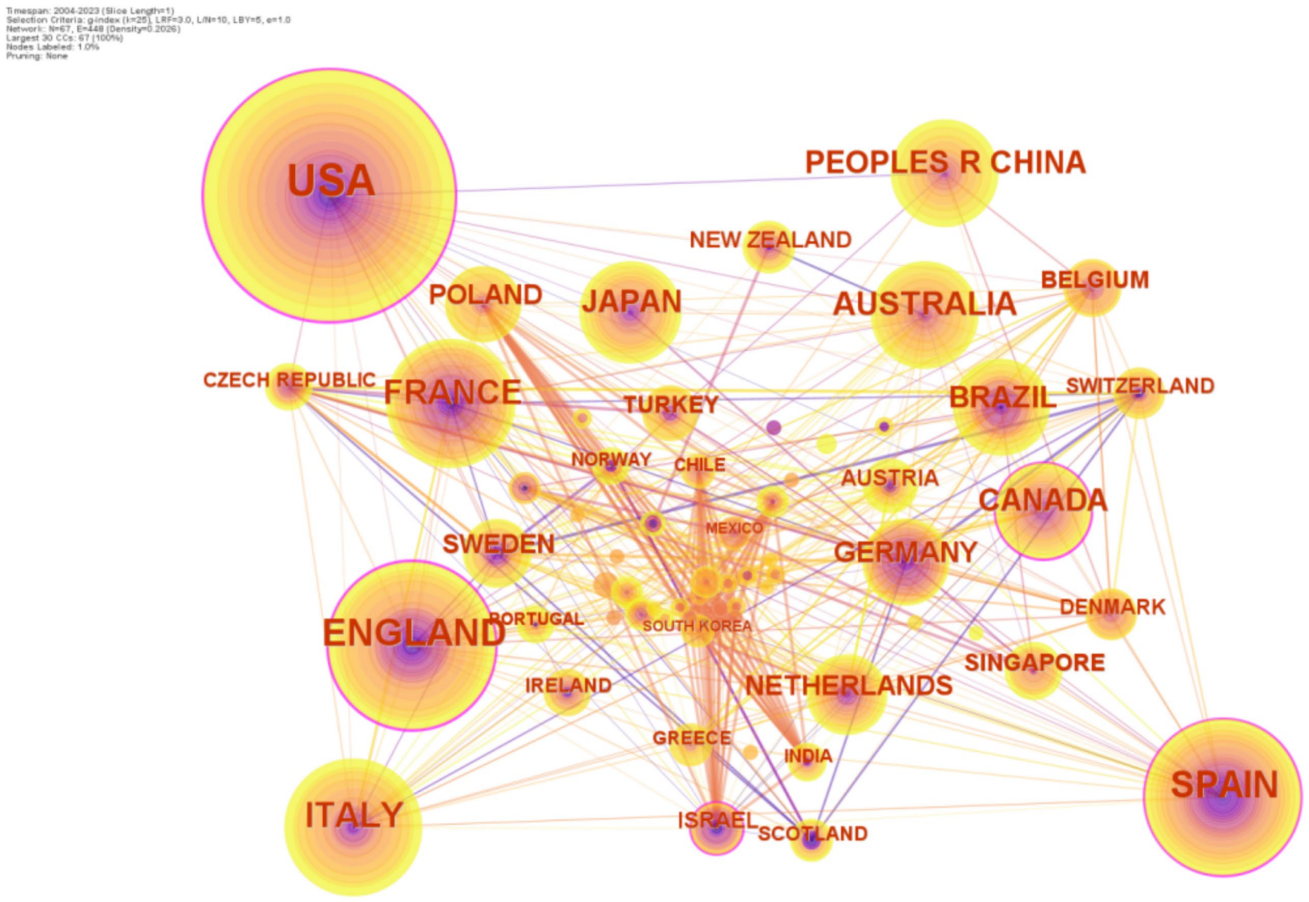
The national cooperation map of HEN from 2004 to 2023.

From 2004 to 2023, a total of 67 countries/regions participated in the publication of 1,113 documents on HEN. CiteSpace generated a distribution map of countries/regions, which showed 112 nodes and 880 links (Figure 3). Table 2 lists the top 10 countries/regions in terms of the number of papers published in the HEN research field. The United States leads with 302 papers, making it the scientific center of the HEN field worldwide. Following the United States are the United Kingdom with 135 papers, Spain with 118, Italy with 97, and France with 82. In terms of centrality, the United Kingdom ranks first with a score of 0.26, followed by the United States (0.21), Spain (0.11), Canada (0.11), and Australia (0.06), indicating close collaborative relationships among them. Countries/regions with central positions play a significant role in HEN research. Despite the UK having a lower number of publications, its role in research is significant. China, with 60 publications, has a centrality score of 0, indicating that despite a substantial number of publications, China’s connectivity and influence on the network map are not significant. Future research in this field should deepen, promoting interdisciplinary and interfield cooperation, enhancing researchers’ innovative thinking and global communication skills, effectively improving the research level of HEN in China.

**Table 2:** TOP10 countries in the HEN study.

Ranking	County	Count	Centrality
1	USA	302	0.21
2	ENGLAND	135	0.26
3	SPAIN	118	0.11
4	ITALY	97	0.01
5	FRANCE	82	0.01
6	AUSTRALIA	62	0.06
7	PEOPLES R CHINA	60	0.00
8	JAPAN	58	0.00
9	CANADA	53	0.11
10	BRAZIL	45	0.02

### 3.4 Analysis of institutions

**Figure 4:**
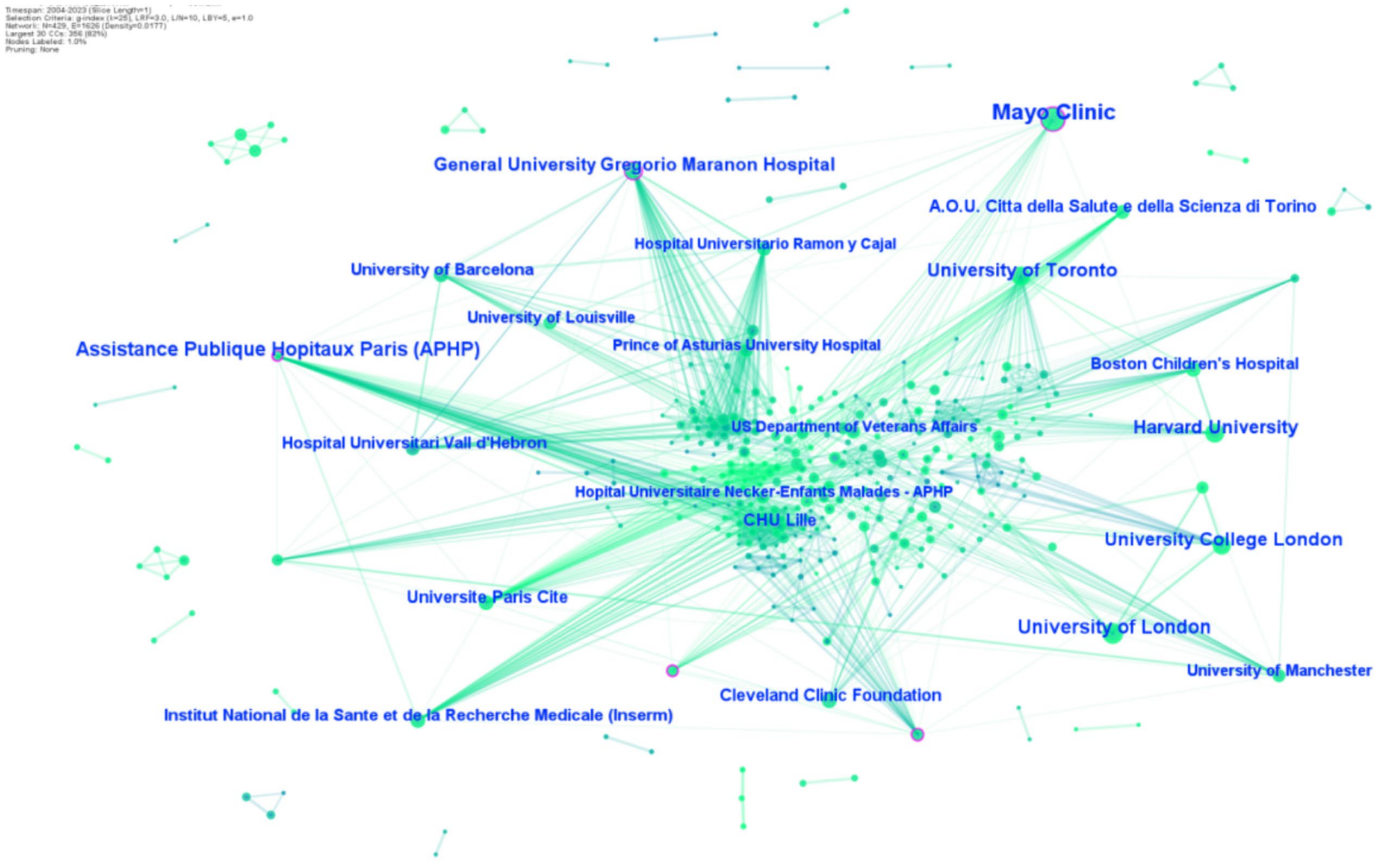
Map of institutions on HEN from 2004 to 2023.

In the field of HEN, research has been conducted by 86 institutions. CiteSpace generated a distribution map featuring 429 nodes and 1,626 links (Figure 4), where each circle represents a research institution. The size of the circle correlates positively with the volume of publications by the institution. The links between circles indicate collaborations between institutions, with the color of the link reflecting the start of the collaboration and the thickness indicating the strength of the partnership. Table 3 lists the top 10 institutions by number of papers published in the HEN research field, with the top five being: Mayo Clinic (41 publications), Assistance Publique Hopitaux Paris (APHP) (26 publications), University of London (24 publications), University of Toronto (22 publications), and Harvard University (22 publications). Visual analysis identified three institutions with a centrality ≥0.1: Mayo Clinic, Assistance Publique Hopitaux Paris (APHP), and General University Gregorio Maranon Hospital. These institutions have been long engaged in HEN-related research and possess strong academic capabilities and international influence.

**Table 3:** TOP10 institutions in the HEN study.

Ranking	Institution	Count	Centrality
1	Mayo Clinic	41	0.1
2	Assistance Publique Hopitaux Paris (APHP)	26	0.1
3	University of London	24	0.02
4	University of Toronto	22	0.08
5	Harvard University	22	0.03
6	University College London	21	0.04
7	General University Gregorio Maranon Hospital	19	0.17
8	CHU Lille	17	0.01
9	Universite Paris Cite	17	0.07
10	Cleveland Clinic Foundation	17	0.06

### 3.5 References analysis

Highly cited documents have laid the foundation for research and accelerated the development of the field (17). Table 4 lists the top 10 cited documents. Among these top 10 documents, 5 are guidelines, 3 are research articles, and 2 are reviews. The top 5 cited documents, in order, are by Bischoff SC (cited 57 times) (2), Mundi MS (cited 51 times) (18), Gramlich L (cited 34 times) (19), Pironi L(cited 33 times) (20), Arends(cited 29 times) (21).

**Table 4:** The top 10 cited articles in fertility concerns.

Ranking	Literature	Count	Centrality	Year
1	ESPEN guideline on home enteral nutrition. Clinical Nutrition,	57	0.01	2020
2	Prevalence of Home Parenteral and Enteral Nutrition in the United States	51	0.04	2017
3	Home Enteral Nutrition: Towards a Standard of Care	34	0.06	2018
4	ESPEN Guidelines on Chronic Intestinal Failure in Adults	33	0.28	2016
5	ESPEN guidelines on nutrition in cancer patients	29	0.11	2017
6	Blenderized Tube Feeding Use in Adult Home Enteral Nutrition Patients: A Cross-Sectional Study	23	0.03	2015
7	ESPEN guidelines on definitions and terminology of clinical nutrition	22	0.01	2017
8	ASPEN Safe Practices for Enteral Nutrition Therapy	22	0.04	2017
9	Guidelines for the Provision and Assessment of Nutrition Support Therapy in the Adult Critically Ill Patient: Society of Critical Care Medicine (SCCM) and American Society for Parenteral and Enteral Nutrition (A.S.P.E.N.)	21	0.01	2016
10	Use of Blenderized Tube Feeding in Adult and Pediatric Home Enteral Nutrition Patients	21	0.05	2017

### 3.6 Analysis of keywords

#### 3.6.1 Keyword co-occurrence

**Figure 5:**
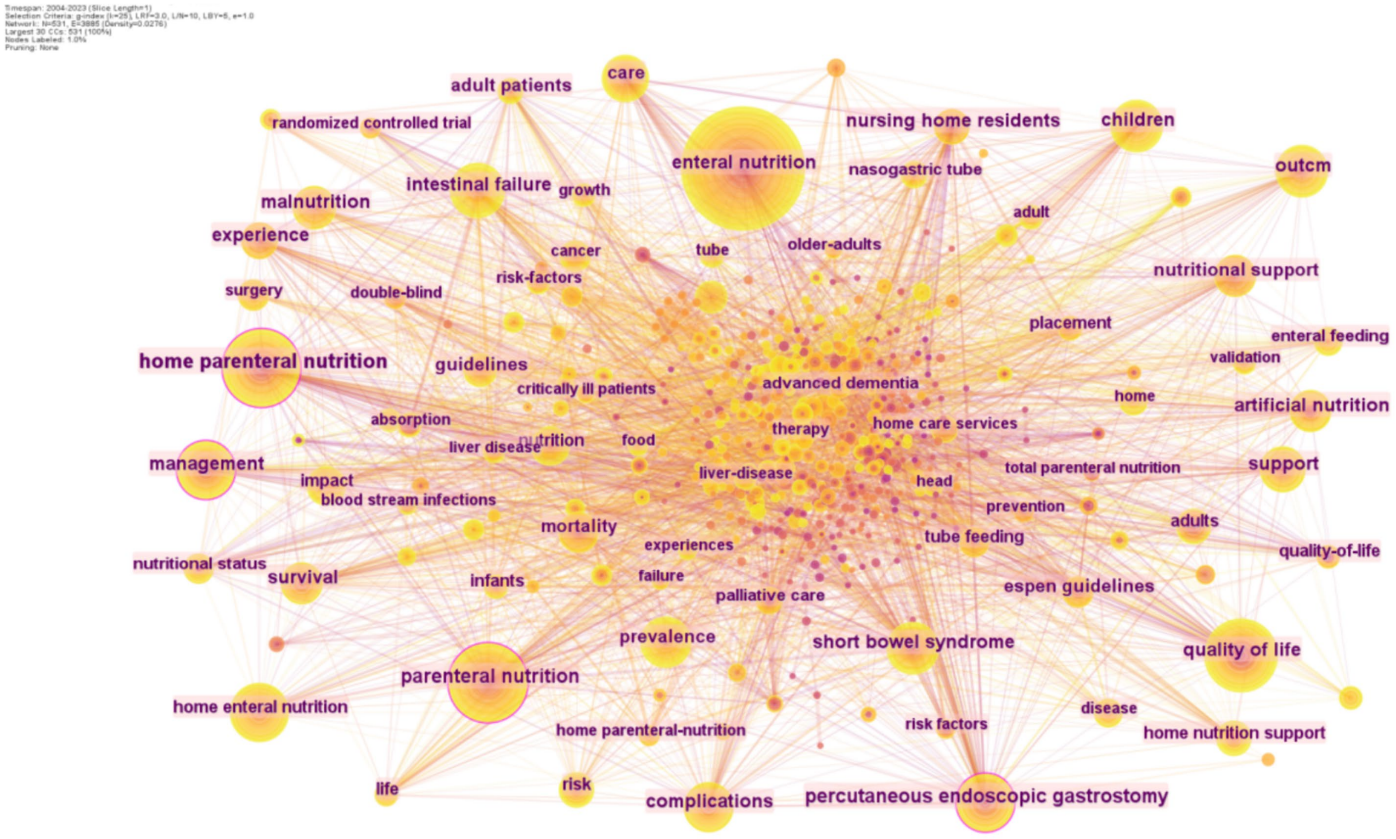
The map of co-cited reference on HEN from 2004 to 2023.

Analyzing keyword frequency within a specific research area can significantly aid researchers in identifying the hot topics and trends of that area. By selecting keywords as the node type, researchers can generate a visualization map of keyword co-occurrence. The co-occurrence analysis of keywords from 2004 to 2023 in the WoSCC database for HEN (Home Enteral Nutrition) revealed that the most frequently occurring keyword is “enteral nutrition,” followed by “parenteral nutrition” (Table 5). The keywords with the highest centrality are “home parenteral nutrition” (0.18), “gastrostomy tube” (0.12), and “management” (0.11). From this analysis, it can be inferred that the management of HEN is currently a prominent research hotspot (Figure 5).

**Table 5:** The top 10 keywords cited articles in the HEN study.

Ranking	Keyword	Count	Centrality
1	Enteral nutrition	424	0.09
2	Parenteral nutrition	190	0.12
3	Home parenteral nutrition	189	0.18
4	Quality of life	155	0.08
5	Home enteral nutrition	125	0.04
6	Percutaneous endoscopic gastrostomy	120	0.12
7	Children	108	0.07
8	Management	97	0.11
9	Intestinal failure	92	0.05
10	Outcome	89	0.06

#### 3.6.2 Keyword burst

**Figure 6:**
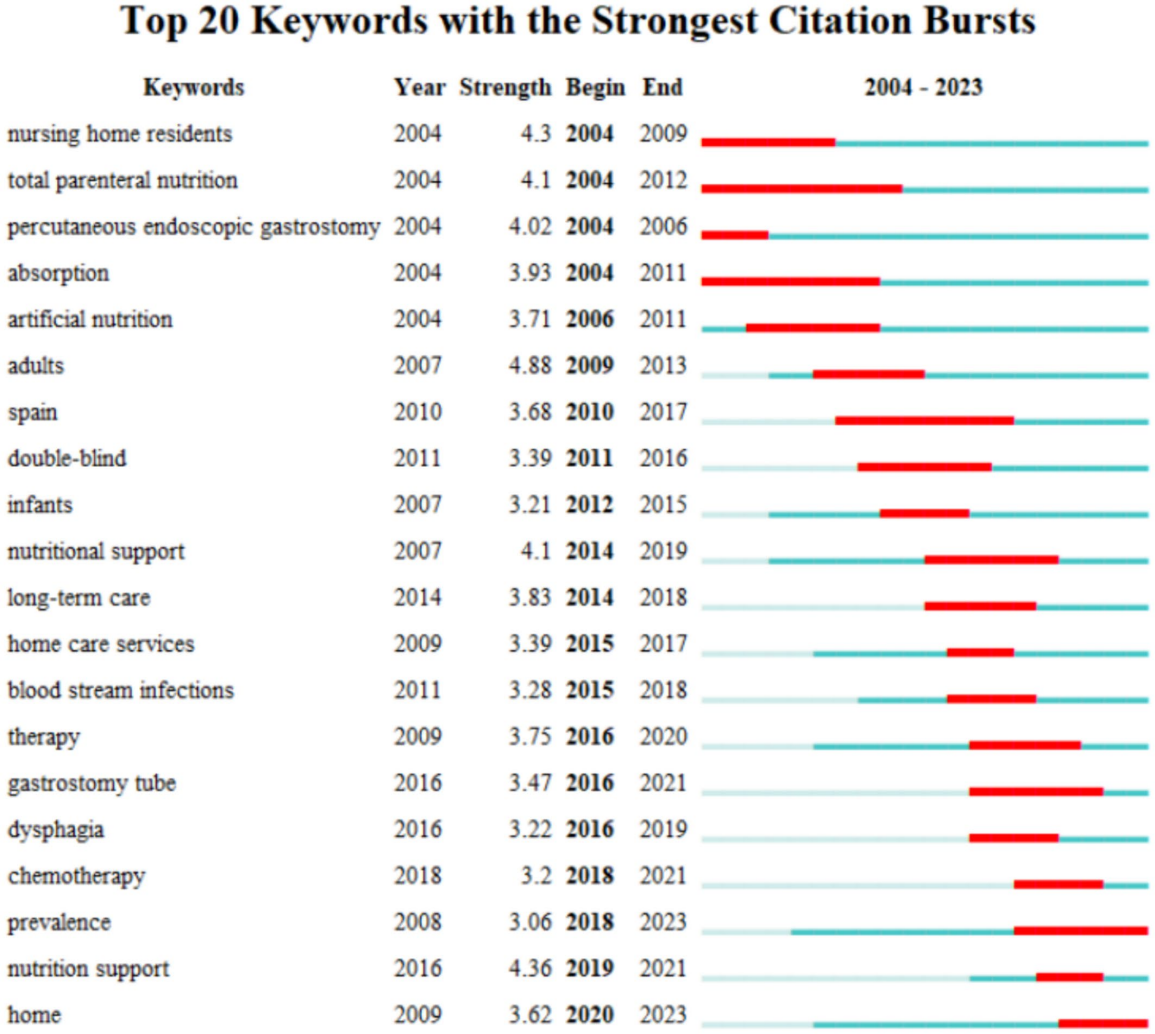
Map of keyword with the strongest citation bursts on the HEN study from 2004 to 2023.

The concept of “burst keywords” as applied in CiteSpace refers to keywords that have been detected within a certain time frame and to some extent can reflect the dynamics and cutting-edge trends of a research field. The keyword burst graph for HEN from 2004 to 2023 is represented by a horizontal line for the time axis, with the red line indicating the period of strongest intensity (Figure 6). Keywords such as total parenteral nutrition, absorption, adults, infants, Spain, double-blind study, home care services, long-term care, and gastrostomy tubes have been persistent subjects of research, reaching the peak of their burst intensity in various time periods, but have since been replaced by other keywords. Nutritional support has been a research hotspot since its emergence in 2014 and continued to be so until 2021. Recent burst keywords include gastrostomy tubes, dysphagia, chemotherapy, and the graphic analysis predicts that home and morbidity are likely to be research directions worthy of attention in the coming years.

## 4 Discussion

The current state and level of research in a field can be deduced by examining annual publication volumes and their distribution trends. Based on bibliometric analysis, this study conducts a visual examination of research related to the HEN field, revealing rapid development over the past two decades. In the WoSCC database, the number of publications in 2023 has increased nearly fivefold compared to 2004, showing a consistent annual growth trend. This indicates that the field is subject to widespread research interest and possesses a promising development trajectory. There are discernible research connections among nations, institutions, and scholars, resulting in some collaborative networks that have garnered attention; however, these connections are relatively limited. Overall, European countries have the densest cooperation networks, indicating more frequent exchanges and collaborations. In Asia, China and Japan have a higher number of publications, but the research tends to be more independent, with a notable lack of collaboration.

### 4.1 Research hotspots and frontiers

In the 1970s, the United States pioneered the concept of home nutrition support (HNS), providing home nutrition support services for cancer patients who still required treatment and rehabilitation after discharge (22). This initiative brought the nutritional issues of patients’ post-discharge into focus. In recent years, HEN has become more widespread due to its simplicity, convenience, and alignment with the physiological characteristics of patients. However, the disadvantages of HEN include the difficulty of technical operations, the risk of infection and complications, psychological burden on patients and their families, challenges in monitoring and management, inadequate social support, cost issues, and challenges in treatment compliance. Therefore, in recent years, an increasing number of scholars have begun to focus on the management of HEN, which has become a current and future research hotspot in the field. Researchers are forming multidisciplinary teams to provide comprehensive and specific home enteral nutrition support for patients at home, aiming to minimize the occurrence of accidents during enteral nutrition in a home setting (23, 24). Further research goals include conducting standardized management training to help patients and caregivers better self-manage HEN support, thereby reducing the incidence of adverse events.

### 4.2 Nutritional techniques in home care and patient-centered outcomes

In the realm of HEN, the range of nutritional techniques has expanded from simple syringe feeding to complex assistance with enteral nutrition pumps. While the safety of these methods in home setting has been well established, the patient-centered longitudinal outcomes still require further elucidation (25). Recent longitudinal studies suggest that patient involvement in enteral nutrition needs to start with improving compliance and reducing the incidence of complications, leading to better long-term health outcomes (26). The psychological impact of HEN on patients is also worthy of attention, as autonomy and comfort in the living environment are key factors affecting overall health. Furthermore, the development of telemedicine has begun to bridge the gap between home care and clinical support, offering patients remote monitoring and real-time feedback on their nutritional status. The convergence of technology and patient-centered care paves the way for a new model of HEN, where personalized care plans not only meet the nutritional needs of patients but also enhance their quality of life.

### 4.3 Challenges and progress of HEN in different populations

Enteral nutrition, while a critical life-sustaining intervention, faces unique challenges when implemented across diverse populations. In pediatrics, it’s imperative to use formulations that not only promote growth and enhance compliance but also implement strategies to prevent complications such as aspiration pneumonia (27). In contrast, elderly patients often present with comorbidities that necessitate enteral nutrition, such as dysphagia related to stroke or neurodegenerative diseases that impair swallowing (28). Advances in this field have led to the development of disease-specific formulas and feeding protocols aimed at minimizing aspiration risks and ensuring adequate nutritional absorption. For patients undergoing treatment for malignancies, the role of enteral nutrition in supporting treatment tolerance and facilitating recovery remains a critical area of research. Here, enteral nutrition as a supplement to chemotherapy or radiation therapy represents an increasingly researched area, with studies highlighting its potential benefits in maintaining body weight and muscle mass, critical for patient recovery and survival. The challenge now lies in tailoring home enteral nutrition to not only meet the medical conditions of patients but also consider their sociocultural backgrounds, which significantly affect their dietary habits and acceptance of medical interventions (29).

### 4.4 Evolution of long-term HEN management and support practices

Recent research is continuously reshaping the landscape of home enteral nutrition. A pivotal element in this evolution is the shift towards a holistic approach that encompasses not only nutritional aspects but also the emotional and psychological support required by patients receiving HEN. The management of HEN has transitioned from a passive to an active stance, with a focus shifting from treatment to the prevention of nutritional deficiencies (30). For instance, the development of advanced predictive tools and algorithms now enables early identification of patients at risk of malnutrition, allowing for proactive nutritional support. Furthermore, increasingly sophisticated enteral formulas, designed to mimic the natural components of food and enhance gastrointestinal tolerance, represent a significant advancement. Current practices in home enteral nutrition typically involve regular follow-ups by dietitians and healthcare providers to ensure that the nutritional plan remains aligned with the patient’s evolving health status (29). The aim is to establish a dynamic, adaptable enteral nutrition strategy that can be customized according to changes in a patient’s condition, thereby ensuring the sustainability and effectiveness of long-term nutritional therapy.

## 5 Conclusion

Over the past two decades, research into home enteral nutrition has deepened significantly, with rapid advancements in the field of management and a solid foundation of scholarly work. The safety of home enteral nutrition and enhancing patient compliance emerge as critical areas for researchers to focus on, suggesting promising directions for future studies. Telemedicine technologies have blurred the lines between hospital and home care, prompting a need for in-depth exploration into how these innovations can be better utilized to serve a broader patient base. Researchers worldwide should leverage their unique characteristics and strengths, foster international collaboration, and achieve scientific breakthroughs. Such efforts aim to promote the wider adoption of home enteral nutrition across various settings, ultimately improving patient quality of life.

## 6 Limitation

This study represents the first bibliometric analysis of HEN. Only the WoSCC database was included as the source of records. Subsequently, to better present the analysis and ensure the quality of the included literature, we conducted a quality assessment of the included documents, limiting our selection to articles and reviews published in English. This may introduce some selection bias. CiteSpace, developed by Professor Chaomei Chen of Drexel University, was the analytical tool used in this study. Due to the nature of the software, there may be biases in some subtle data, and the interpretation of standard visual maps may not be entirely consistent, which could also impact the results of data analysis. Our goal is to improve the accuracy and interpretation of data in subsequent research.

## Author contributions

SQ: Conceptualization, Data curation, Project administration, Validation, Writing – original draft, Writing – review & editing. QC: Conceptualization, Data curation, Software, Supervision, Validation, Writing – original draft. JH: Formal analysis, Supervision, Validation, Writing – review & editing. DX: Conceptualization, Software, Writing – review & editing. KH: Data curation, Software, Writing – review & editing. YS: Formal analysis, Validation, Writing – original draft. QH: Formal analysis, Supervision, Validation, Writing – review & editing. YP: Software, Supervision, Validation, Writing – original draft, Writing – review & editing.
